# *Didelphis albiventris*: an overview of unprecedented transcriptome sequencing of the white-eared opossum

**DOI:** 10.1186/s12864-019-6240-x

**Published:** 2019-11-15

**Authors:** Íria Gabriela Dias dos Santos, Tiago Antônio de Oliveira Mendes, Gerluza Aparecida Borges Silva, Amanda Maria Sena Reis, Cláudia Barros Monteiro-Vitorello, Patricia Dayane Carvalho Schaker, Roberto Hirochi Herai, André Brait Carneiro Fabotti, Luiz Lehmann Coutinho, Erika Cristina Jorge

**Affiliations:** 1 Departamento de Morfologia, Instituto de Ciências Biológicas, Universidade Federal de Minas Gerais, Belo Horizonte, Minas Gerais Brazil; 2 Departamento de Bioquímica e Biologia Molecular, Universidade Federal de Viçosa, Viçosa, Minas Gerais Brazil; 3 Departamento de Genética, Escola Superior de Agricultura Luiz de Queiroz, Universidade de São Paulo, Piracicaba, São Paulo Brazil; 4 Graduate Program in Health Sciences, School of Medicine, Pontifícia Universidade Católica do Paraná (PUCPR), Curitiba, Paraná, Brazil; 5 Centro Federal de Educação Tecnológica de Minas Gerais, Belo Horizonte, Minas Gerais Brazil; 6 Departamento de Zootecnia, Escola Superior de Agricultura Luiz de Queiroz, Universidade de São Paulo, Piracicaba, São Paulo Brazil

**Keywords:** *Didelphis albiventris*, White-eared opossum, RNA-seq, Transcriptome, Newborn, Postnatal

## Abstract

**Background:**

The white-eared opossum (*Didelphis albiventris*) is widely distributed throughout Brazil and South America. It has been used as an animal model for studying different scientific questions ranging from the restoration of degraded green areas to medical aspects of Chagas disease, leishmaniasis and resistance against snake venom. As a marsupial, *D. albiventris* can also contribute to the understanding of the molecular mechanisms that govern the different stages of organogenesis. Opossum joeys are born after only 13 days, and the final stages of organogenesis occur when the neonates are inside the pouch, depending on lactation. As neither the genome of this opossum species nor its transcriptome has been completely sequenced, the use of *D. albiventris* as an animal model is limited. In this work, we sequenced the *D. albiventris* transcriptome by RNA-seq to obtain the first catalogue of differentially expressed (DE) genes and gene ontology (GO) annotations during the neonatal stages of marsupial development.

**Results:**

The *D. albiventris* transcriptome was obtained from whole neonates harvested at birth (P0), at 5 days of age (P5) and at 10 days of age (P10). The de novo assembly of these transcripts generated 85,338 transcripts. Approximately 30% of these transcripts could be mapped against the amino acid sequences of *M. domestica*, the evolutionarily closest relative of *D. albiventris* to be sequenced thus far. Among the expressed transcripts, 2077 were found to be DE between P0 and P5, 13,780 between P0 and P10, and 1453 between P5 and P10. The enriched GO terms were mainly related to the immune system, blood tissue development and differentiation, vision, hearing, digestion, the CNS and limb development.

**Conclusions:**

The elucidation of opossum transcriptomes provides an out-group for better understanding the distinct characteristics associated with the evolution of mammalian species. This study provides the first transcriptome sequences and catalogue of genes for a marsupial species at different neonatal stages, allowing the study of the mechanisms involved in organogenesis.

## Background

Marsupials are one of the three large modern groups of mammals and are considered the closest external group to eutherian mammals (placentals). They comprise a group of 350 extant species found in the Americas, mostly in South America and Australasia [1].

The most intriguing characteristic of marsupials is related to the way they develop: after a very short period of intrauterine development, they are born with only a few early developed structures (including the external nostrils, the mouth and the forelimbs), while all other structures are still in early stages of development. In general, lactation plays a major role in the development of marsupial joeys [2], whereas the placenta is short-lived, and most marsupial placentas are either non-invasive, as in the tammar wallaby [3, 4], or invasive in only the last few days of pregnancy, as in the South American grey short-tailed opossum [3, 5]. Immediately after birth, neonates climb into the marsupium pouch, where each attaches to one of the mother’s teats, and a complex system of lactation is established while the main organogenesis stages of development take place [1–12]. In the pouch, marsupial neonates are in an altricial developmental stage equivalent to E10–12 of mouse embryos or 10 weeks of human development [6].

Marsupials can be considered an excellent model for understanding the mechanisms that govern cellular differentiation during organogenesis due their close phylogenetic relationship to eutherians and the availability of neonates in the pouch (overcoming the difficulty of access when studying in utero development). For instance, different aspects of neocortex development, expansion and evolution have recently been revealed using embryos and neonates of the grey short-tailed opossum (*Monodelphis domestica*) and the tammar wallaby (*Macropus eugenii*) as models [13, 14]. Studies using grey short-tailed opossum neonates revealed new insights into bronchioalveolar [15] and chondrocranial development in mammals [16]. Morphological and morphometric study of the skin of marsupial neonates suggested the participation of this organ in gaseous exchange on the basis of the investigation of species such as the eastern quoll (*Dasyurus viverrinus*), grey short-tailed opossum (*Monodelphis domestica*), southern brown bandicoot (*Isoodon obesulus*), long-nosed bandicoot (*Perameles nasuta*), brush-tail possum (*Trichosurus vulpecula*), koala (*Phascolarctos cinereus*), long-nosed potoroo (*Potorous tridactylus*), brush-tailed rock wallaby (*Petrogale penicillata*), red-necked pademelon (*Thylogale thetis*) and black-striped wallaby (*Macropus dorsalis*) [17]. Marsupials, especially didelphids, have been employed as models for the study of mammalian evolution [18–20].

Such important biological characteristics can be discovered in marsupials with strong support from genomic and/or transcriptomic sequences available in public databanks. The present work adds to a growing number of new marsupial genome and transcriptome datasets that have been released in the last few years, which have mainly been developed to the study of the marsupial placenta, lactation and immune system, in species such as the grey short-tailed opossum (*Monodelphis domestica*) [21, 22], the Tasmanian devil (*Sarcophilus harrisii*) [23, 24], the tammar wallaby (*Macropus eugenii*) [12, 25, 26], the koala (*Phascolarctos cinereus*) [27–29], the long-nosed bandicoot (*Perameles nasuta*) [30, 31], the fat-tailed dunnart (*Sminthopsis crassicaudata*) [32], the monito del monte (*Dromiciops gliroides*) [33], and the Tasmanian tiger (*Thylacinus cynocephalus*) [34].

Based on these previous findings and genomic data availability limitations, the goal of this work was to sequence the transcriptome of the white-eared opossum (*Didelphis albiventris*) during the first days of its postnatal development. This is the first transcriptome obtained from a South American marsupial species during distinct stages of neonatal development. *D. albiventris* undergoes intra-uterine development for 13 days, in which the last ~ 3 days are dependent on a non-invasive placenta [35]; after birth, the neonates remain with the mother for ~ 100 days depending on lactation [36, 37]. For this work, we used neonates harvested at birth and at 5 and 10 days old.

These marsupials are widely distributed in South America (including Cerrado, Caatinga and Pantanal areas) [38–42], and they have been used as model organisms to understand human infections such as Chagas disease [43, 44] and leishmaniasis [45]. Cáceres [46] suggested that *D. albiventris* is an important agent as a seed spreader. The seeds of many plant species (including pioneers) may aid in the restoration of degraded environments after they are eaten by opossums, as they remain viable after passing through the intestine [46]. *D. albiventris* and other didelphids are resistant to the venom of snakes such as *Bothrops* spp., *Crotalus durissus* and *Lachesis muta* [47–49]. They are also resistant to intoxication by millipedes [50], which are toxic to many vertebrates. Our group has been trying to establish *D. albiventris* as a model for understanding odontogenesis stages during development. This opossum exhibits complete heterodont dentition that is closer to that of humans than is that of rodents, the typical model for these studies [8, 51–55]. We can characterize the morphological stages of early tooth development (dental lamina, bud, cap, and bell stages) in this species [8, 51–55]. However, the use of sequences from *M. domestica* (the closest evolutionary relative) to develop molecular approaches to study *D. albiventris* has not been successful.

Analysis of the *D. albiventris* transcriptome has generated genetic information for this promising species as a new model organism for studies on the regulatory molecular mechanisms of organogenesis, providing a better understanding of marsupial species contributing to their preservation and supporting evolutionary developmental biology research [56].

## Results

### RNA-seq and transcriptome assembly of *D. albiventris*

This work generated transcriptomes (RNA-seq) of the white-eared opossum during the first days of its postnatal development. The analysis of all RNA-seq samples was performed; on average, over 47.5 million raw reads per library/biological replicate were obtained for each postnatal stage (Table 1) with a 100 bp paired-end fragment length. Using the Trinity tool, 85,338 transcripts were assembled, considering a minimum fragment overlap of 35 bp and contigs with a length of at least 300 bp.

**Table 1:** RNA-seq data

Samples type	Total number of reads	Sample description
P0	50,523,432	Biological replicate 1 for P0
	51,490,816	Biological replicate 2 for P0
	47,805,980	Biological replicate 3 for P0
P5	50,433,754	Biological replicate 1 for P5
	47,708,526	Biological replicate 2 for P5
	47,014,722	Biological replicate 3 for P5
P10	46,287,998	Biological replicate 1 for P10
	52,374,156	Biological replicate 2 for P10
	33,968,928	Biological replicate 3 for P10

#### Sequence composition among biological replicates

**Fig. 1:**
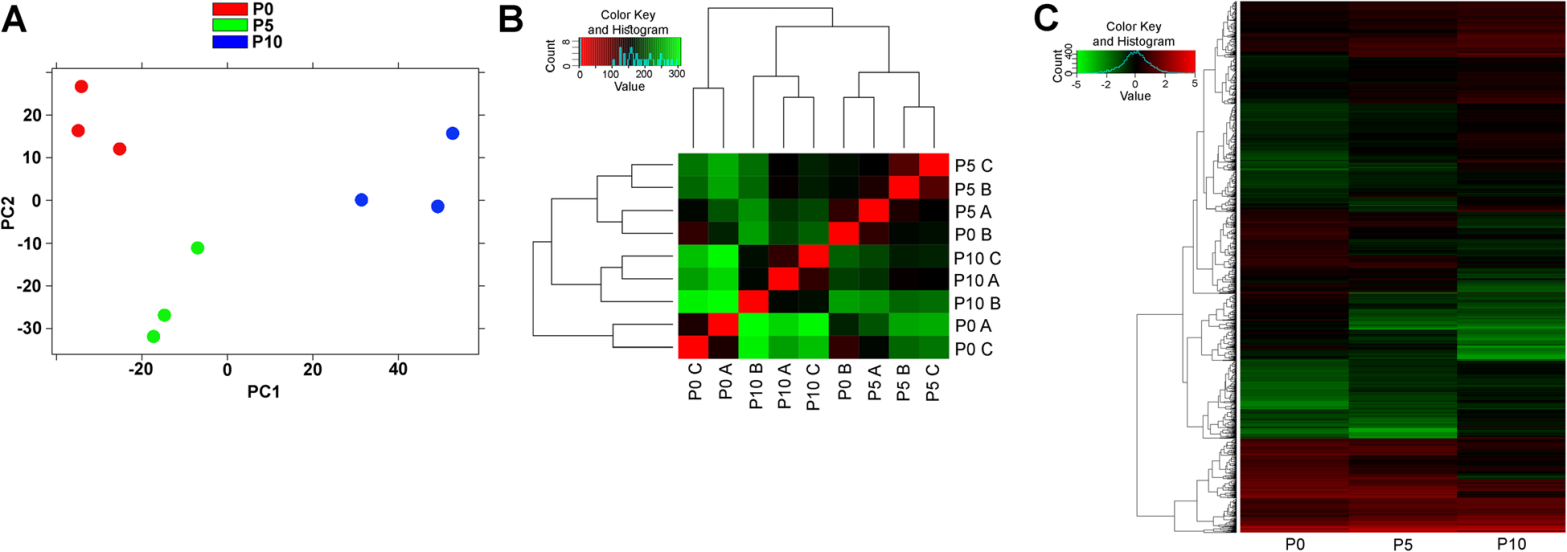
Bioinformatic analysis of RNA-seq data**.** Homogeneity of the biological triplicates determined by PCA (**a**) and the colourmap matrix **b** based on transcript expression values between P0, P5 and P10. In the colourmap matrix, the differences between the samples are indicated as follows: green dots represent the most different samples, and the red dots represent the least different samples (**b**). Colourmap matrix based on transcripts expressed at the three stages, with expression levels ranging from green (low values) to red (high values) (**c**)

Principal component analysis (PCA) showed that the biological replicates for each developmental stage could be grouped together, forming 3 distinct clusters corresponding to the different sets of biological conditions (Fig. 1a).

The colourmap matrix constructed to show the sample-to-sample distances confirmed that the three biological replicates for each developmental stage were very similar to each other, except for one P0 sample that showed similarity to the P5 samples (see “P0 B” in Fig. 1b). The expression levels of each transcript throughout the three developmental stages were represented as a colourmap matrix, allowing the observation that the expression of several transcripts did not change considerably across samples P0, P5 and P10. It was also possible to observe that some transcripts varied between the samples, showing either increasing or decreasing expression across the sample types (Fig. 1c).

#### Differentially expressed genes between opossum postnatal stages

**Fig. 2:**
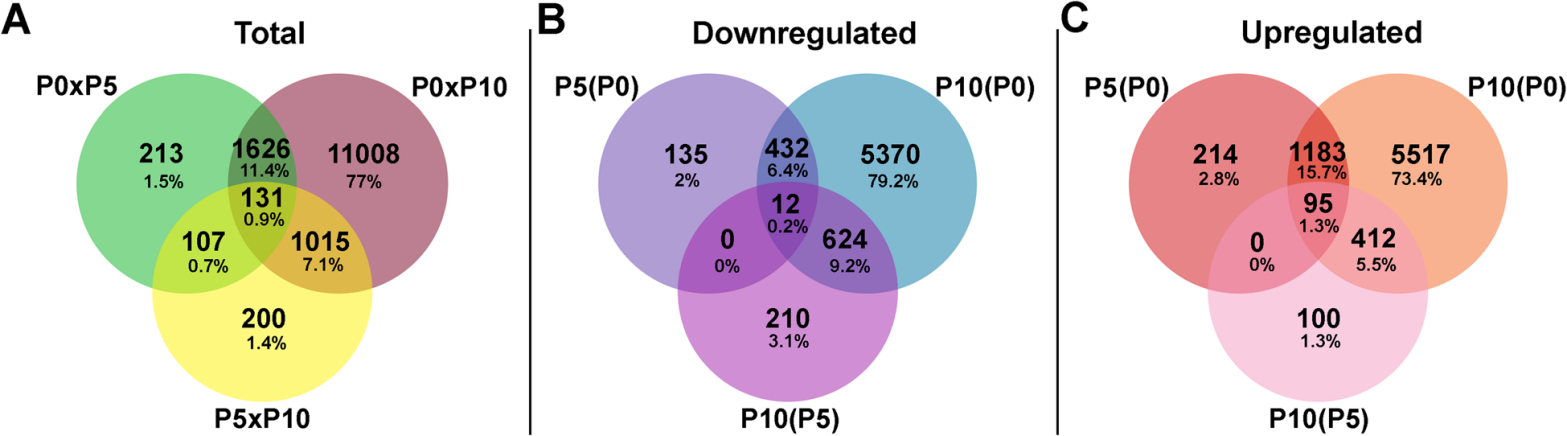
Venn diagrams**.** Venn diagram for differentially expressed (*p* < 0.05) transcripts between P0 and P5, P5 and P10, and P0 and P10 (**a**). Venn diagram for differentially expressed (p < 0.05) transcripts showing downregulation [log_2_(fold change) < − 1; **b**] or upregulation [log_2_(fold change) > 1; **c**]. The transcripts are described as exhibiting an increase or decrease in the expression level at the older stage compared to the younger stage. Images were generated using Venny 2.1

We identified 14,300 differentially expressed (DE) transcripts between the P0, P5 and P10 biological stages. Among the total DE transcripts, 2077 transcripts were DE between stages P0 and P5, 13,780 between stages P0 and P10, and 1453 between stages P5 and P10 (Fig. 2a). Additionally, 213 of these transcripts were DE exclusively between stages P0 and P5 and 200 between P5 and P10 (Fig. 2a). The most striking difference was observed between P0 and P10, as 11,008 DE transcripts were identified between these stages (Fig. 2a). Additionally, 131 transcripts were found to be DE between all analysed developmental stages (Fig. 2a).

Among the 14,300 DE transcripts, 579 were found to be downregulated (Fig. 2b) and 1492 to be upregulated (Fig. 2c) at P5 in relation to P0; 6438 were downregulated (Fig. 2b), and 7207 upregulated (Fig. 2c) at P10 in relation to P0; and 846 were downregulated (Fig. 2b), and 607 were upregulated (Fig. 2c) at P10 in relation to P5.

We also identified a few transcripts that were exclusively expressed in each of the analysed stages: 109 transcripts were exclusively expressed at P0, 149 at P5 and 237 at P10 (Additional file 3).

### Amino acid sequence similarities between *D. albiventris* and *M. domestica*

The BLASTX analysis of the *D. albiventris* translated nucleotide sequences against the amino acid sequences from *M. domestica* showed that the sequences that exhibited 30% coverage were more likely to present higher identity values and less likely to present lower identity values (Additional file 1).

### *D. albiventris* nucleotide sequence similarities to other marsupial transcriptomes

BLASTN analysis of the *D. albiventris* assembled nucleotide sequences against available marsupial transcriptomes showed higher identity values between the *D. albiventris* and *M. domestica* (90%) or *S. harrisii* (90%) sequences compared to those of *P. cinereus* (20%) and *M. eugenii* (20%) (Additional file 2).

### Transcriptome assembly validation

#### Validation by RT-qPCR

We validated four DE transcripts from our RNA-seq analysis by RT-qPCR. The selected transcripts were associated with genes involved in enriched biological processes related to haematopoietic cells (HMGB3) [57, 58], platelet agglutination (APOH) [59, 60], erythrocytes (HBZ) [61], and the eye lens (CRYGB) [62, 63].

**Fig. 3:**
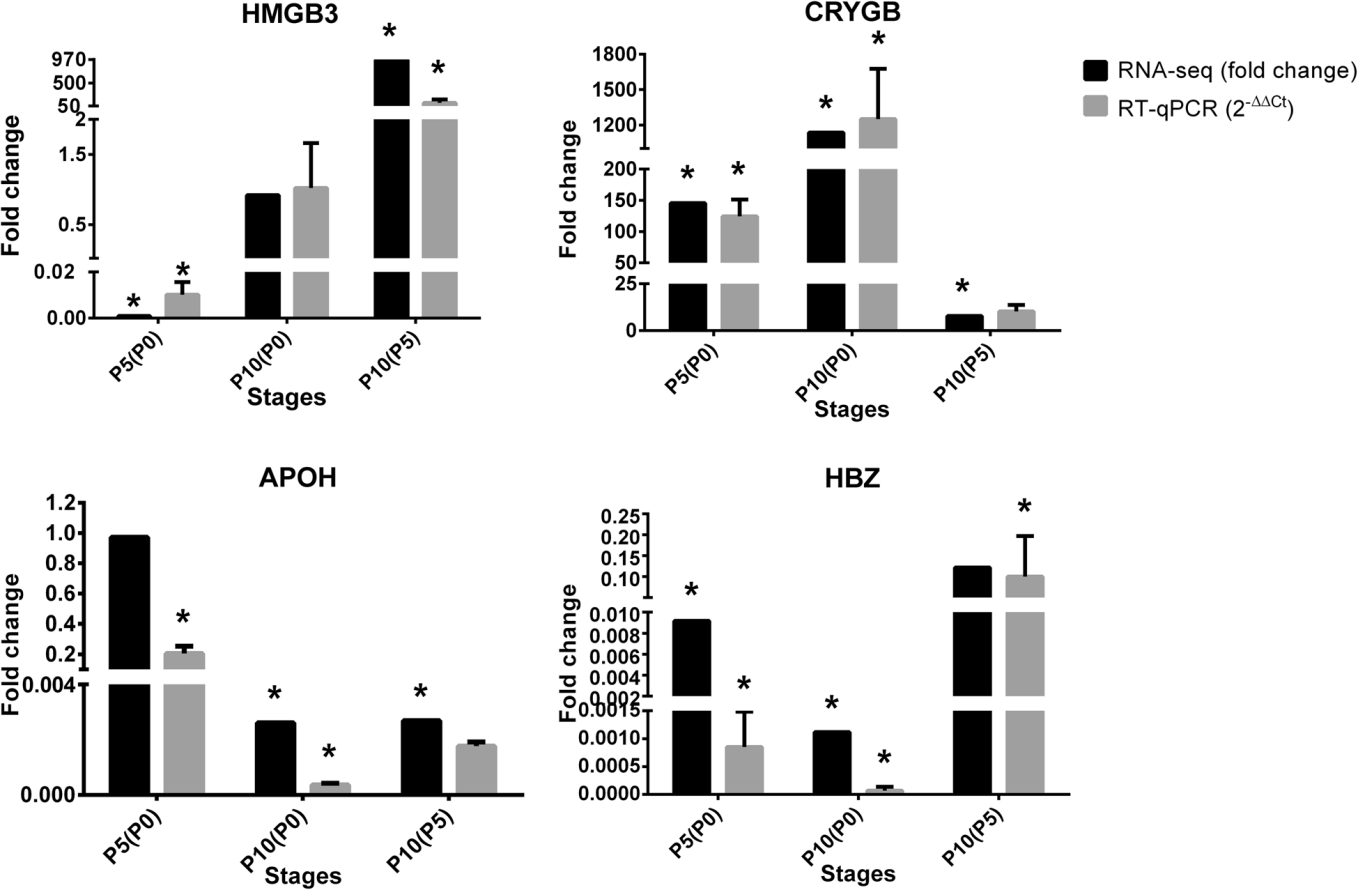
Validation of the RNA-seq expression profiles by RT-qPCR. To evaluate the validity of the Illumina sequencing results and confirm the observed patterns of expression, a subset of four differentially expressed genes among the most upregulated and downregulated genes between the stages was selected and evaluated by RT-qPCR analysis. The expression patterns indicate agreement between the two methods. Relative expression was evaluated between the developmental stages, and P0 was used as a calibrator. *Asterisk*, *p* < 0.05; *X-axis*, developmental stage comparisons (P0 vs P5, P0 vs P10, P5 vs P10); *Y-axis*, fold change (2^-ΔΔCt^ values for RT-qPCR and fold change values for RNA-seq) in mRNA levels; *bars*, mean; *error bars*, standard deviation (SD)

Although there were a limited number of genes to validate these data, we observed total concordance in the direction of expression but not in the magnitude of the expression change between the RNA-seq and RT-qPCR results (Fig. 3). In the RNA-seq data (Additional file 4), HMGB3 was found to be downregulated at P5 compared to its expression at P0, while it was upregulated at P10 compared to P5. CRYGB was upregulated at P5 relative to P0 and at P10 relative to P0; APOH was downregulated at P10 relative to P5; and HBZ was downregulated at P10 relative to P0.

#### *Validation by* in situ *hybridization (ISH)*

A ~ 400 bp fragment of opossum titin mRNA could also be amplified and cloned. Sections of P0, P5 and P10 tissues were selected to show the presence of titin mRNA in skeletal muscles by using the ISH method (Additional file 5). The RNA-seq data showed higher expression levels of titin transcripts at later postnatal stages.

Our results revealed the expected muscle-specific staining for titin in all postnatal stages. At P0 (Additional file 5 b and c) and P10 (Additional file 5 i), the tongue muscle fibres were strongly stained by ISH. At P5, the ventral skeletal muscle of the body wall (Additional file 5 d) and longitudinal and transversal sections of limb skeletal muscle (Additional file 5 f) were strongly stained by ISH. The diaphragm (Additional file 5a, e and h) showed moderate staining of the skeletal muscle. At P10, the heart showed moderate staining (Additional file 5 g), and the extrinsic muscles of the eye showed strong staining (Additional file 5 J).

### GO pathway enrichment analysis

The enriched GO terms for the DE transcripts were generally related to the cell cycle, metabolism and tissue survival as well as the development of systems such as the (i) immune system, (ii) blood tissue, (iii) hearing, (iv) vision, (v) digestion, (vi) the excretory system, (vii) the nervous system, and (viii) the limbs (Tables 2 and 3).

**Table 2:** Enriched DE transcripts between P0 and P5

Tissue, system or organ	GO term	Gene name	Encoded protein – expression and function
Immune system	Antigen processing and presentation	CD8A	Mediation of efficient cell-cell interactions within the immune system
	Mucosal immune response	PIGR	Facilitation of the transcytosis of soluble polymeric isoforms of immunoglobulin A and immune complexes
	Thymic T cell selection; T cell selection	CD74	Association with MHC class II and regulation of antigen presentation for the immune response
Blood tissue	Erythrocyte differentiation	AHSP	Molecular chaperone that binds specifically to free alpha-globin and is involved in haemoglobin assembly
		DYRK3	Acts as a negative regulator of EPO-dependent erythropoiesis
		KLF1	Transcription regulator of erythrocyte development that probably serves as a general switch factor during erythropoiesis
Hearing	Cochlea development	HES1	Expressed in the organ of Corti during its terminal differentiation
		PTK7	Guides stereocilia orientation
		SOBP	Regulates the cellular fate and standardization of the organ of Corti
Vision	Visual perception	AIPL1	Expressed during rod and cone development
		CRYBA1, CRYBA4, CRYBB1, CRYBB2	Crystallins are required for the development and maintenance of the transparency of the eye lens
		GJC1	Expressed in retina bipolar cells and retinal ganglion cells during development
Digestion	Gastric acid secretion	SLC9A4	Required for normal levels of gastric acid secretion, secretory membrane development, parietal cell maturation and/or differentiation and, at least secondarily, for chief cell differentiation
		GAST	Gastrin is a hormone that stimulates the secretion of hydrochloric acid by the gastric mucosa, which results in gastrin formation inhibition. Also acts as a mitogenic factor for gastrointestinal epithelial cells
Excretory system	Metanephric nephron development	IRX1	Expressed and required during different stages of pronephros development. Additionally, it is crucial in the regionalization and patterning of tissues and organs during metazoan development
Nervous system	Midbrain development; regulation of astrocyte differentiation; regulation of glial cell differentiation	GPR37L1	Expressed in neurons and glia
	Midbrain development	MSX1	Related to the development of the midline structure of the forebrain, expressed in the spinal cord
Limbs	Embryonic hindlimb morphogenesis	FGF4	Expressed in the thoracic and pelvic limbs and the apical ectodermal ridge of the limb bud
		MSX1	Encodes transcription factors that are crucial for limb development

**Table 3:** Enriched DE transcripts between P5 and P10

Tissue, system or organ	GO term	Gene name	Encoded protein – expression and function
Immune system	Complement activation	C1S	Serine protease that is a major constituent of human complement subcomponent C1
		C4BPA, C4BPB	Controls the classical pathway of complement activation
		C6	Component of the complement cascade
	Mucosal immune response	PLA2G1B	Secreted member of the phospholipase A2 (PLA2) class of enzymes produced by the pancreatic acinar cells
		RAB17	The small Rab GTPases are key regulators of intracellular membrane trafficking from the formation of transport vesicles to their fusion with membranes
Blood tissue	Blood coagulation	APOH	Binds to cardiolipin
		FGB, FGG	Plays a role in blood clotting and platelet aggregation
		HRG	Binds to numerous ligands and modulates immunity, vascularization and coagulation processes
		PDGFA	Regulation of embryonic development, cell proliferation, cell migration, survival and chemotaxis
		PROC, PROZ	Regulation of blood coagulation
	Fibrin clot formation	APOH	Binds to cardiolipin
		FGB, FGG	Important for blood clotting and platelet aggregation
	Complement and coagulation cascade	F7	Initiates the extrinsic pathway of blood coagulation
		F9	Participates in the intrinsic pathway of blood coagulation
		PLG	Plasminogen is activated by proteolysis and converted to plasmin and angiostatin
	Erythrocyte differentiation	AHSP	Prevents the harmful aggregation of alpha-haemoglobin during normal erythroid cell development
		KLF1	Transcription regulator of erythrocyte development that probably serves as a general switch factor during erythropoiesis
Digestion	Fat digestion and absorption; glycerolipid metabolism	MOGAT3	Catalyses the formation of diacylglycerol from 2-monoacylglycerol and fatty acyl-CoA
Excretory system	Regulation of kidney development	FAT4	Plays a role in the orientation of cell divisions and tubule elongation during kidney development

**Fig. 4:**
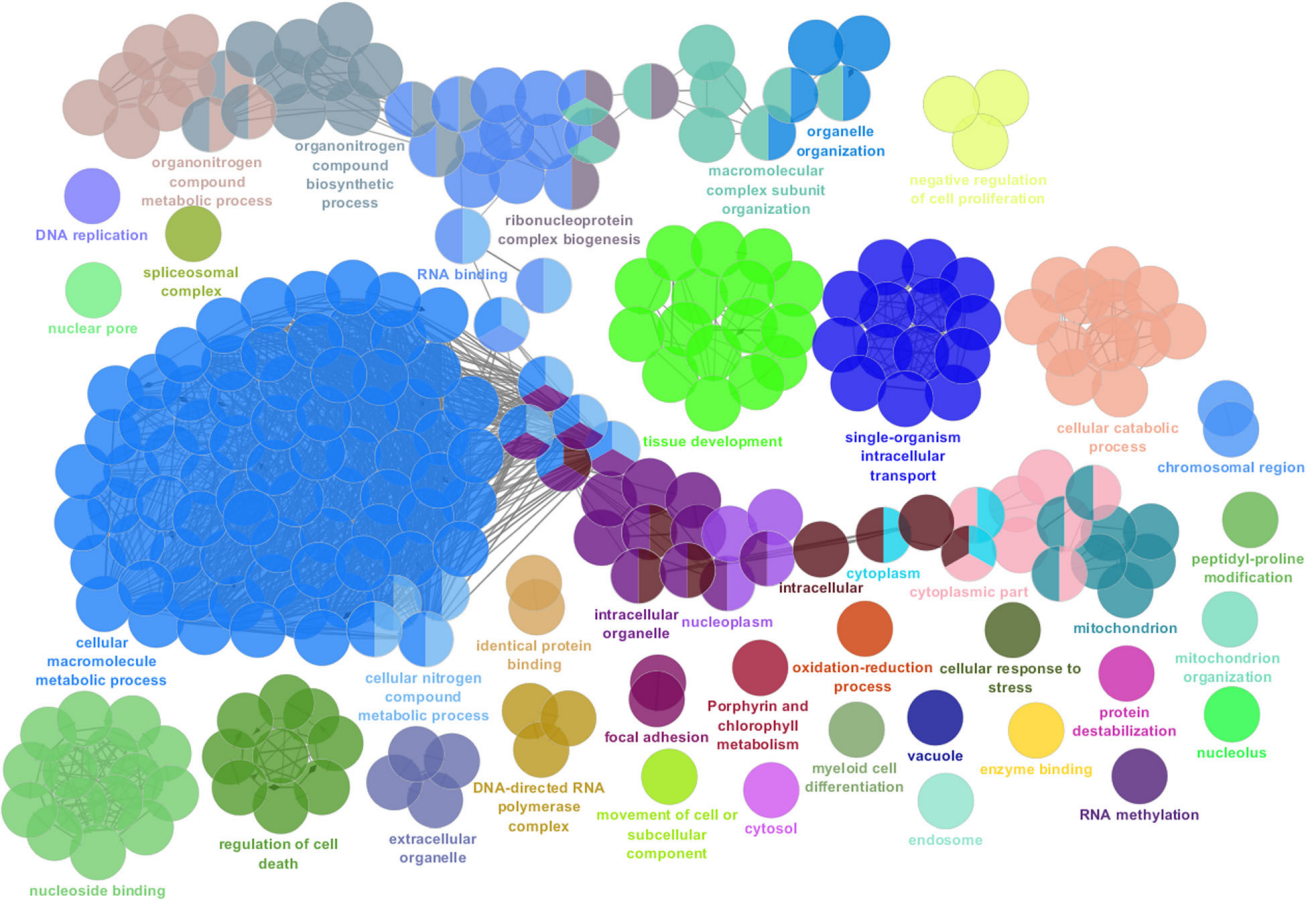
Analysis of enriched GO functional groups of expressed genes between the developmental stages. Functionally grouped networks of enriched categories for expressed genes between the developmental stages, annotated for the cellular component, biological process, molecular function, and immune system process KEGG and GO terms. All genes were considered in this analysis, including differentially expressed and non-differentially expressed genes. Only the most significant term in the group is labelled. GO terms are represented as nodes. The edges connecting the nodes are based on the Kappa statistic (Kappa score threshold of 0.4), which measures the overlap of shared genes between terms. The right-sided hypergeometric test was used for statistical inference, and the Benjamini-Hochberg method was applied in *p* value correlation (*p* < 0.05)

**Fig. 5:**
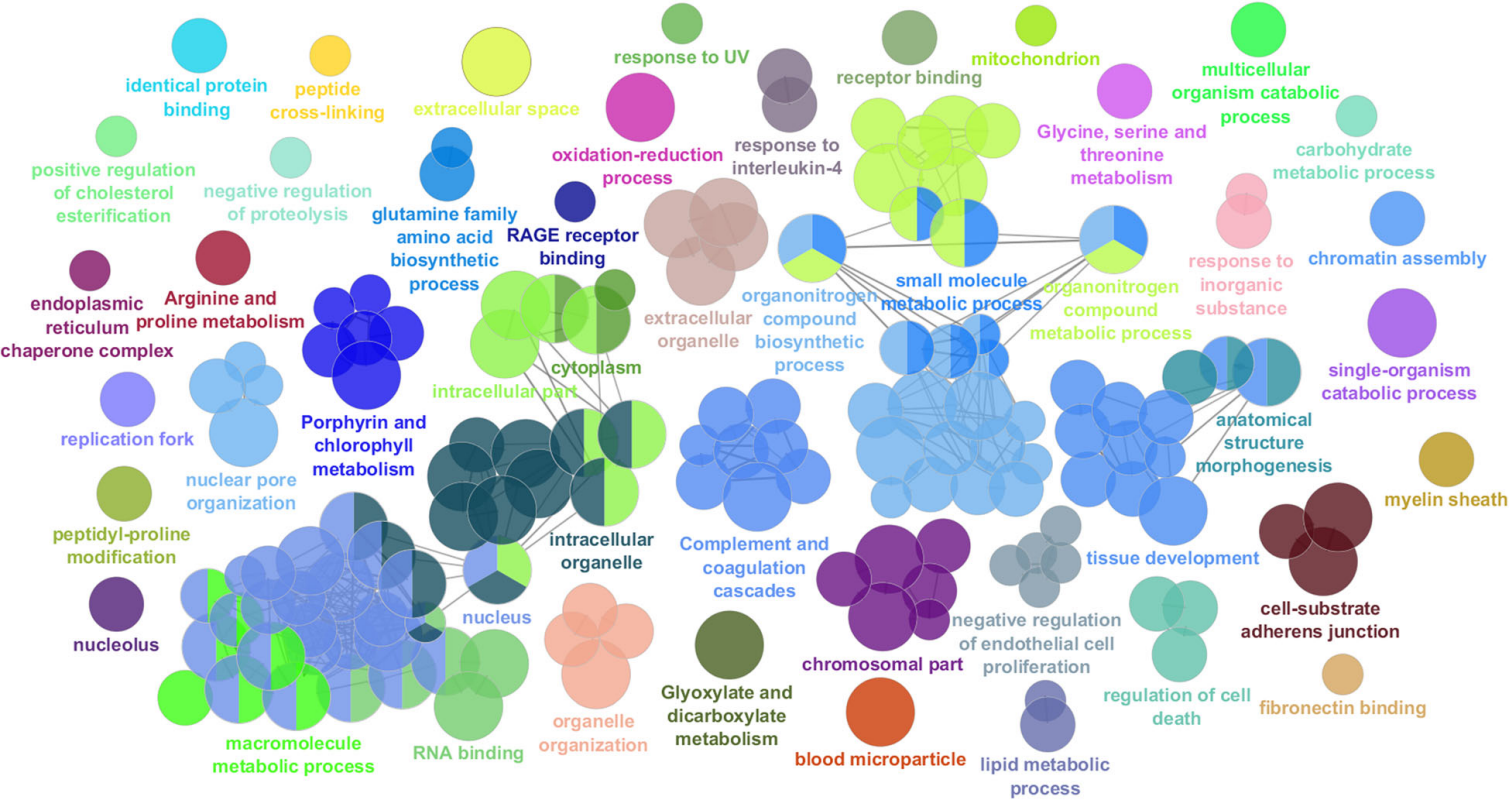
Analysis of enriched GO functional groups of differentially expressed genes between the developmental stages**.** Functionally grouped networks of enriched categories for differentially expressed genes between the developmental stages, annotated for the cellular component, biological process, molecular function, and immune system process KEGG and GO terms. Only the most significant term in the group is labelled. GO terms are represented as nodes, and the node size represents the term enrichment significance. The edges connecting the nodes are based on the Kappa statistic (Kappa score threshold of 0.4), which measures the overlap of shared genes between terms. The right-sided hypergeometric test was used for statistical inference, and the Benjamini-Hochberg method was applied for p value correlation (*p* < 0.05)

**Fig. 6:**
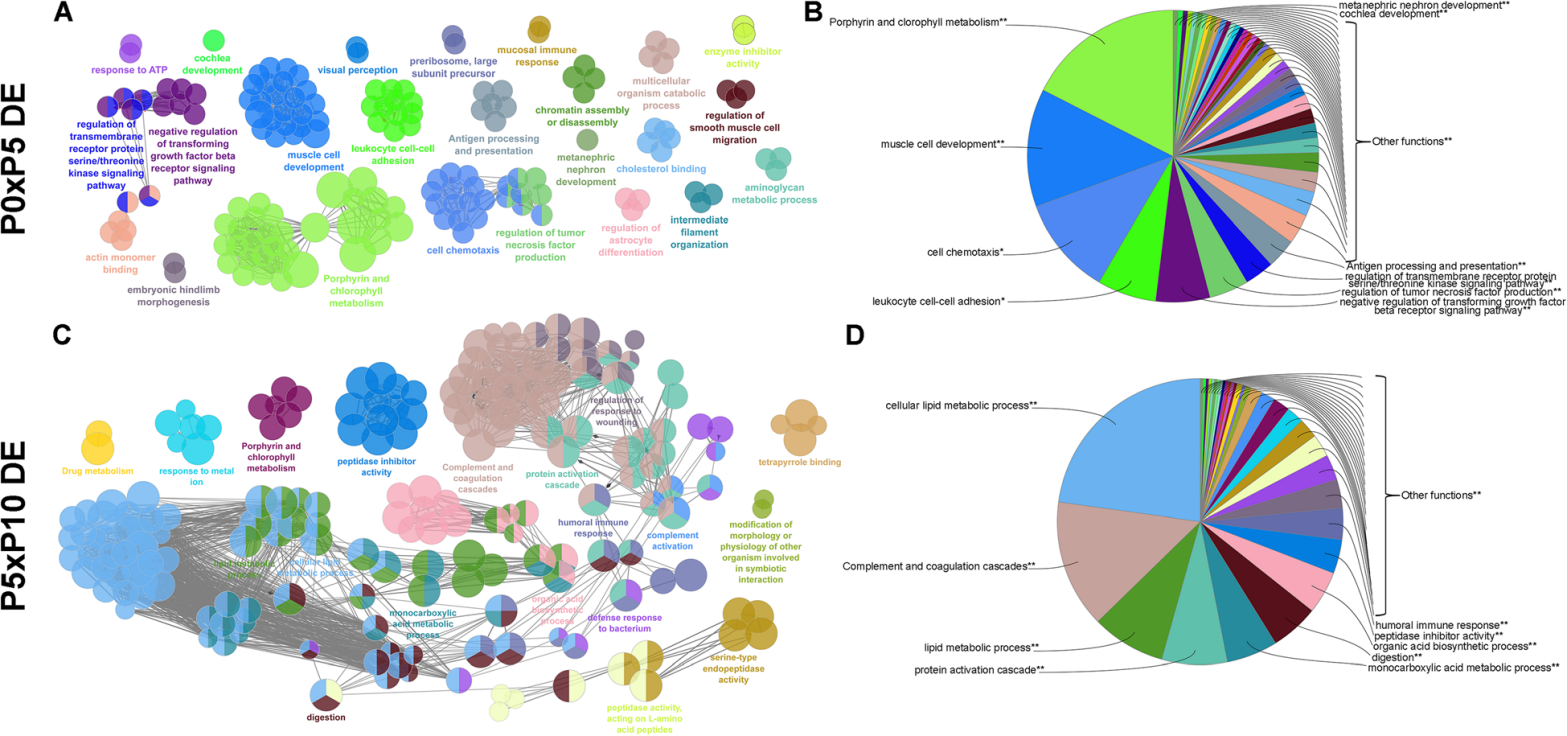
Analysis of enriched GO functional groups of DE genes at P0xP5 and P5xP10. Functionally grouped networks of enriched categories for DE genes between P0 and P5 (**a**) and between P5 and P10 (**b**), annotated for the cellular component, biological process, molecular function, and immune system process KEGG and GO terms. Only the most significant term in the group is labelled. GO terms are represented as nodes, and the node size represents the term enrichment significance. The edges connecting the nodes are based on the Kappa statistic (Kappa score threshold of 0.4), which measures the overlap of shared genes between terms. The right-sided hypergeometric test was used for statistical inference, and the Benjamini-Hochberg method was applied for p value correlation (*p* < 0.05)

**Fig. 7:**
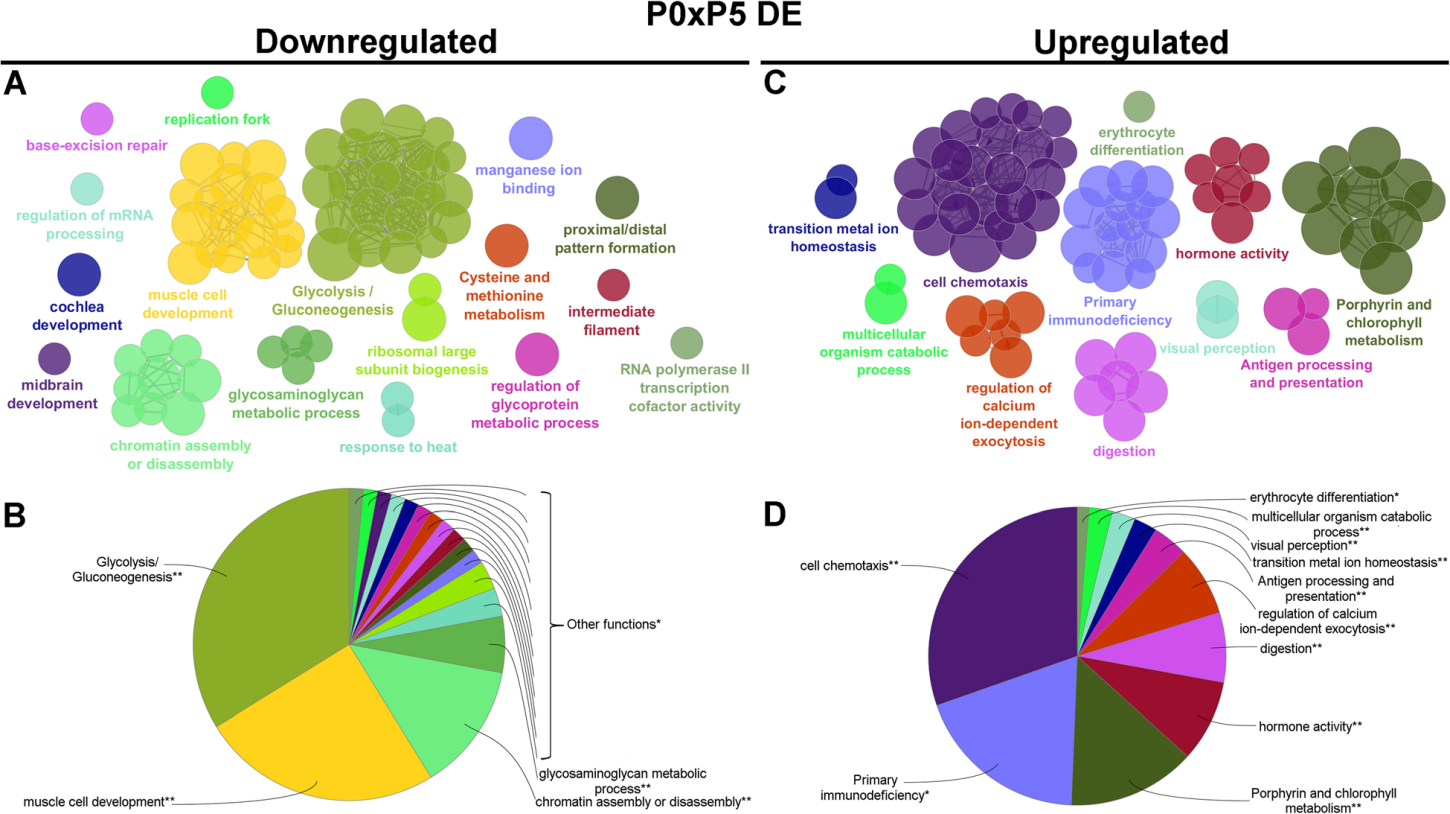
Analysis of enriched GO functional groups of downregulated and upregulated DE genes at P0xP5**.** a functionally grouped network of enriched categories was generated for the downregulated (**a** and **b**) and upregulated (**c** and **d**) differentially expressed genes at P5 in relation to P0. The most significant term in each functional group is represented. The cellular component, biological process, molecular function, and immune system process KEGG and GO terms were selected for this analysis. The right-sided hypergeometric test was used for statistical inference, and the Benjamini-Hochberg method was applied for p value correlation (*p* < 0.05)

**Fig. 8:**
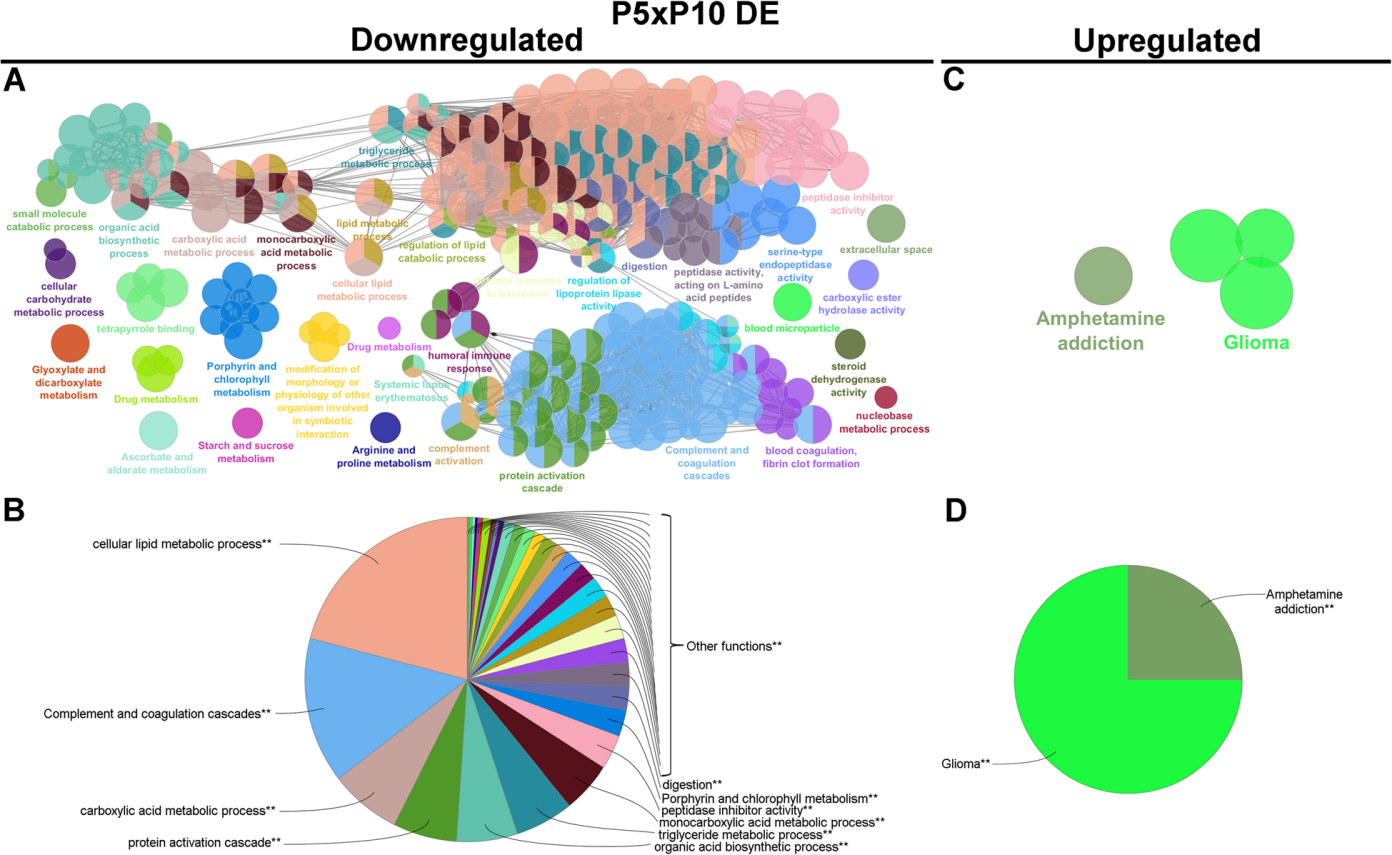
Analysis of enriched GO functional groups of downregulated and upregulated DE genes at P5xP10**. a** functionally grouped network of enriched categories was generated for the downregulated (**a** and **b**) and upregulated (**c** and **d**) differentially expressed genes at P10 in relation to P5. The most significant term in each functional group is represented. The cellular component, biological process, molecular function, and immune system process KEGG and GO terms were selected for this analysis. The right-sided hypergeometric test was used for statistical inference, and the Benjamini-Hochberg method was applied for p value correlation (*p* < 0.05)

The most significant GO terms are shown in Figs. 4 to 8 and Additional file 6 for all 85,338 transcripts expressed among the three postnatal stages (Fig. 4); for the 14,300 DE transcripts expressed among the three postnatal stages (Fig. 5); for the 2077 DE transcripts between P0 and P5 (Fig. 6a-b); for the 579 downregulated DE transcripts (Fig. 7a-b) and 1492 upregulated DE transcripts (Fig. 7c-d) at P5 relative to P0; for the 1453 DE transcripts between P5 and P10 (Fig. 6c-d); and for the 846 downregulated DE transcripts (Fig. 8a-b) and 607 upregulated DE transcripts (Fig. 8c-d) at P10 relative to P5.

## Discussion

In this work, we sequenced the transcriptomes of whole neonates of the white-eared opossum (*Didelphis albiventris*) in three postnatal developmental stages to reveal the mechanisms of development in this South American marsupial species. To the best of our knowledge, this is the first RNA-seq transcriptome assembly available for this species. Although some studies have investigated the transcriptomes of certain tissues of marsupial neonates [15, 64, 65], this is the first differential expression analysis of whole marsupial individuals in distinct neonatal development stages. The generated database, composed of 85,338 transcripts, could be used to reveal the mechanisms of late tissue specification and differentiation in a marsupial model, contributing to the understanding of molecular phylogenetic aspects of mammalian evolution.

### Transcriptome resources

Our transcriptome database was obtained from three *Didelphis albiventris* siblings per developmental stage (P0, P5 and P10), which is considered a sufficient number of biological replicates for a reliable quantitative inferential analysis [66]. On average, 47.5 million reads were obtained per library/biological replicate of each postnatal stage. Although the general homogeneity of the sequenced biological triplicates is supported by the PCA clustering results, Euclidian distance-based analysis, represented by a colourmap matrix, suggested that a single P0 replicate slightly deviated from the other samples at the same stage. Discordance in gene expression patterns between siblings may occur due to epigenetic factors [67, 68] such as marsupium environment conditions and lactation.

The coverage of just 30% between the amino acid sequences of *D. albiventris* and its closest evolutionary relative sequenced to date, *M. domestica*, revealed the importance of the opossum transcriptome for understanding the biology of this species and the evolution of the marsupial group. Many reads from unannotated transcripts may be lost, and various multi-mapping reads are observed because of reads falling within exons that are shared by different transcript isoforms of the same gene [66].

Considering the similarity of mapped nucleotide sequences (BLASTN), the assembled *D. albiventris* transcripts showed more similarity at the nucleotide level with sequences of *M. domestica* (grey short-tailed opossum) and, surprisingly, *S. harrisii* (Tasmanian devil) than with *P. cinereus* (koala) and *M. eugenii* (tammar wallaby). Taken together, these results corroborate the importance of generating molecular information for a new South American marsupial species, as its phylogenetically closest sequenced relative shows low levels of sequence similarity. New marsupial phylogenetic relationships can now be revealed from these sequences.

RNA-seq validation is an important step for establishing and verifying biological interpretations that may arise from database analysis [69]. The need for this type of validation has been discussed since the correlation between RNA-seq and RT-qPCR data has been consistently confirmed. Indeed, the present study supported these observations: the quantification of upregulated and downregulated transcripts by RT-qPCR revealed a correlation with the levels and per-stage differences of transcript expression determined by RNA-seq. However, the RT-qPCR assays were performed on the same biological samples used to perform RNA-seq, which supports the validity of bioinformatics DE analysis for the discovery of up- and downregulated molecules between different stages. However, the RT-qPCR assays still present the limitation of not having the power to represent the biological expression of a gene in a population. Other independent biological samples need to be tested to confirm the detected gene expression levels [69].

Transcriptome assembly usually tends to be more complex than genome assembly and to be more challenging in eukaryote organisms than in prokaryotes, especially when it requires de novo assembly [70, 71]. Thus, we decided to validate the assembled transcriptome by ISH as well. Since the young opossum climbs and suckles from the day of its birth [6], we evaluated skeletal muscle development at the studied stages. The strong staining for titin hybridization reflected the presence of mature skeletal muscle fibres in the tongue at P0 and P10, in the body wall at P0, in the limbs at P5, and in the extrinsic muscles of the eye at P10. Additionally, weaker staining for titin hybridization showed a lower degree of maturation in the diaphragm at the three stages and in the heart at P10. The moderate hybridization of titin at P10 in the heart is consistent with reports that most ventricular cardiac muscle cells are fully differentiated by the end of the fifteenth postnatal week [72]. Thus, our data confirmed the specificity of the synthesized probe. The observation of gene expression in a semi-embryonic state model will allow further studies on molecular pathways during mammalian development. Moreover, the muscles of marsupials and other mammals have been recently studied for their role in non-shivering thermogenesis (NST), which is important for endothermic regulation [73]. The elucidation of the *D. albiventris* transcriptome will allow these molecular studies to be extensively performed on opossums.

### The main tissues and organs in the development of the white-eared opossum

The opossum transcriptome obtained during different developmental stages allowed us to establish the first gene expression patterns for different tissues from this marsupial model. As our first dive into this dataset, we decided to only analyse differentially expressed transcripts between each neonatal stage, focusing on the development of tissues that are known to be of particular importance for marsupial functions. Consequently, we could obtain some idea of the most important genic events controlling each stage.

Gene ontology terms associated with the development of the immune system were found to be enriched in our analysis of the *D. albiventris* DE transcripts. In marsupials, the thymus, bone marrow and lymph nodes are still immature at birth [74]. Until the adaptive immune system develops, immune protection of the marsupial newborn is achieved by prenatal transfer of immunoglobulins, antimicrobial compounds within the pouch and immune compounds within the milk [75]. We found a number of “immune”-associated GO terms during the *D. albiventris* developmental stages. The enriched terms grouping the P0 and P5 DE transcripts included “chemokine-mediated signalling pathway”, “intestinal immune network for IgA production”, “leukocyte migration”, “lymphocyte co-stimulation”, “negative regulation of tumour necrosis factor production”, “neutrophil chemotaxis”, “positive regulation of inflammatory response” and “thymic T cell selection”. The enriched terms grouping the P5 and P10 transcripts included “activated T cell proliferation”, “acute inflammatory response”, “cytokine biosynthetic process”, “cytokine secretion”, “defence response to bacterium”, “innate immune response in mucosa”, “neutrophil migration”, “positive regulation of chemokine production” and “positive regulation of interleukin-6 and -8 production”. These data show that *D. albiventris* starts to establish its adaptive immune system at birth, and the main pathways are activated in P5 and P10. Additionally, several genes linked to coagulation, fibrinolysis and complement system components were found to be enriched in our database, similar to what is observed during *M. domestica* early postnatal stages [15]. These components are all interconnected: the complement protein network, an essential component of the innate and humoural immune system [76], participates in several non-inflammatory processes, such as coagulation [77], along with components of the coagulation and fibrinolysis systems [76]. Genes related to erythrocyte differentiation were also observed, such as the erythroid transcription factor KLF1 (also known as EKLF), which is expressed by erythro-myeloid progenitors (EMPs) [78], erythroid cells [79], and megakaryocyte-erythrocyte progenitors (MEPs) [79].

Regarding the use of the primary jaw joint, the marsupial neonate approximates the embryonic condition of mammalian ancestors [80]. Therefore, marsupial neonates have recently come to be considered an excellent resource for studies on the transition from primary to novel jaw articulation through the analysis of inner ear development [81, 82]. In the present study, we found enriched upregulated genes at P0 relative to P5 associated with cochlea development including HES1 (Hairy and Enhancer of Split 1), PTK7 (Tyrosine-Protein Kinase-Like 7) and SOBP (Sine Oculis Binding Protein Homologue), which were categorized under the GO terms “cellular response to retinoic acid” and “cochlea development”. Retinoic acid is indispensable for inner ear development and plays a role in the molecular cascade of hair cell regeneration [83–86]. The differentiation of the inner ear hair cells, located in the cochlea, is negatively regulated by HES1 [87], and the stereocilia orientation in these cells is guided by PTK7 [88, 89]. The SOBP gene is expressed mainly in the cochlear duct, regulating the cellular fate and standardization of the organ of Corti [90, 91]. These data suggest that our gene expression profile of *D. albiventris* at the P0 neonatal stage might include candidate genes that could reveal the molecular mechanisms that determine the development of the cochlea and other inner ear structures.

Eutherian neonates [92] and chicken embryos [93] have been used in studies on eye lens development due the great importance of studying eye-related pathologies that affect humans and can be identified soon after birth, such as congenital cataracts and retinoblastoma [94]. Regarding the importance of understanding eye lens development, our data showed enriched genes that were upregulated at P5 relative to P0 such as CRYBA1, CRYBA4, CRYBB1, and CRYBB2, which were grouped under the following GO terms: “visual perception” and “sensory perception of light stimulus”. The beta-crystalline gene cluster, composed of CRYBA1, CRYBA4, CRYBB1 and CRYBB2, is expressed in the vertebrate eye lens from embryonic to adult life [95]. Additionally, other genes such as AIPL1 (expressed during rod and cone development [96]) and GJC1 (expressed in retina bipolar cells and retinal ganglion cells during development [97]), which were found to be related to enriched GO terms in our results, could be targets for understanding the marsupial visual system.

The gastrointestinal system of postnatal marsupials has been explored, and the results contribute to telling the story of the evolution of this system in vertebrates [98–101]. Studies on marsupial pouch joeys have shown that every phase of milk contains specific factors that trigger phenotypic changes in the neonate stomach [102] and that the milk regulates the microbiota of the neonate intestine [103]. Furthermore, studies indicate that the milk plays a role in accelerating the development and maturation of the stomach [102]. However, the molecular mechanisms involved in these changes during the development of the marsupial stomach are not yet known [102]. Hence, the enriched genes linked to the digestive system identified in this study, such as SLC9A4 and MOGAT3, could help to understand the development of the mammalian gastrointestinal system. These genes are mainly related to stomach, intestine and pancreas functions. SLC9A4 (also known as NHE4) is a Na^+^/H^+^ exchanger that is highly expressed in the basolateral membrane of gastric parietal cells, playing a role in the maintenance of normal levels of gastric acid and gastric epithelial cell differentiation [104]. MOGAT3 (or MGAT3) is expressed in the pancreas and intestines [105, 106] and may play a role in triglyceride absorption [105].

The development of the mammal kidneys is another topic that is still under investigation [107]. Several recent studies have aimed to trace the early stages of renal development to understand the mechanisms involved and implement strategies for the in vitro engineering of kidney tissue replacement (in foetuses and neonates, for example) to develop more precocial, more efficient, and lower-cost therapeutic approaches [108–110]. Among the enriched GO terms identified in the three neonatal stages, the gene IRX1 is correlated with the early stages of kidney development. This gene is required for the formation of intermediate segments of the pronephros [111] and is necessary to maintain the identity of the pronephric territory and define its size [112]. IRX1 is expressed in the proximal tubular segment and the intermediate tubule segments of the pronephros [111, 112] and exhibits an important function in the differentiation of a proximo-medial S-shaped body (SSB) subdomain [113]. Thus, IRX1 could provide valuable information about mammalian kidney development and evolution.

Finally, the rate of brain development in marsupials varies more than in eutherians, and this characteristic might be associated with their longer lactation period [114]. Because the forebrain of marsupials primarily develops postnatally, it is possible to study the brain development of the young in the pouch in stages that are equivalent to human mid-embryogenesis [115]. A study in which in-pouch electroporation was performed using newborns of the Australian marsupial fat-tailed dunnart (*Sminthopsis crassicaudata*) demonstrated that marsupials can be an excellent in vivo model for studying forebrain development and evolution [115]. In the present study, several genes were found to be related to enriched GO pathways involved in nervous tissue development and differentiation, including MSX1 and GPR37L1. While MSX1 is expressed in the spinal cord [116] and is related to the development of the midline structure of the forebrain [117], GPR37L1 is practically restricted to the brain, where it is expressed in neurons and glia [118, 119].

While cranial and adult structures have been receiving attention, there is a gap in the knowledge of the evolution of mammalian post-cranial structures, such as the limbs [120]. A recent morphological study suggests that the marsupial forelimb may place a constraint on evolution due the functional requirements of the crawl to the teat, which would limit morphological variation before and at the time of birth [120]. This peculiar mode of reproduction in marsupials could be responsible for their lower diversity in limb morphology [120]. The authors also emphasize that “adult integration studies may not be indicative of developmental integration” [120], highlighting the importance of studies on embryos and neonates. The enriched genes related to embryonic hindlimb morphogenesis found among opossum developmental stages include FGF4 and MSX1. FGF4 is expressed in the thoracic and pelvic limbs [121] and in the apical ectodermal ridge of the limb bud [122], playing a role in the distal growth of the developing limb bud [121, 123]. MSX1 encodes transcription factors that are crucial for limb development [124].

## Conclusion

In this study, the transcriptomes of whole young animals were sequenced, assembled and compared at three different ages, generating the first comprehensive catalogue of *D. albiventris* transcripts, enabling detailed analysis of the unique attributes of this native Brazilian marsupial. This transcriptomic dataset is a useful resource for molecular genetic studies of the opossum and could be used as an animal model to investigate distinct organ formation and development. As an important model for mammals, the generated dataset provides novel and important dynamic modifications at the molecular level for better understanding of the evolutionary changes in marsupials and other vertebrates.

## Methods

### Macroscopic and microscopic procedures

Postnatal (P) *Didelphis albiventris* (Didelphidae, Marsupialia) individuals at 0, 5 and 10 days of age were harvested from the pouches of pregnant females captured during the breeding season (from June to January) at the Ecologic Station of the Federal University of Minas Gerais (19° 52′ S, 43° 58′ W, Belo Horizonte, MG, Brazil). Six specimens were harvested for each stage and euthanized by decapitation; three of the specimens were randomly selected for RNA-seq procedures and three for histological procedures. Neonates from the same developmental stage were harvested from the same mother and came from the same litter (siblings). All animal procedures were conducted following the instructions of the ethical committee (CEUA/UFMG, ethical approval 220/2008), the Brazilian Institute of Environment and Renewable Natural Resources (IBAMA, SISBIO 27354–2), and the Brazilian laws for the use of animals in scientific experiments.

According to the committee’s instructions, for the removal of the postnatal individuals attached to the mammary papillae, the adult female was immobilized by restraining her cervical region, pelvic limbs and tail. Rotation and traction movements of the embryos were performed to remove the young from the female mammary papillae. These procedures did not require the use of sedation or anaesthetics. Decapitation using a scalpel or steel blade is an immediate, quick procedure, especially in postnatal individuals, without the need for anaesthetics.

After harvesting, the young animals were measured and decapitated, and their bodies were divided into halves along the median plane. All sections of this report adhere to the ARRIVE Guidelines for reporting animal research [125]. A completed ARRIVE guidelines checklist is included in **Checklist S1**.

### In situ hybridization (ISH)

For ISH, oligonucleotides (ATGGCAGCAGGCGGCAGC and TCAGCATCTTAGCAGACATGGGG) were designed using the *D. albiventris* sequence of the titin transcript assembled in this work as a template to allow the amplification of a fragment of ~ 400 bp of its mRNA. The amplified fragment was cloned into the *pGEM-T Easy Vector* (Promega). The antisense RNA probe was synthesized from the SP6 promoter of this clone in the presence of digoxigenin-UTP (Roche).

*D. albiventris* half trunks were fixed in 4% paraformaldehyde (PFA) for 2 days (P0), 4 days (P5), or 7 days (P10) at room temperature (RT) and embedded in paraffin with the anterior surface (for head samples) or the median surface (for body samples) facing the cut plane. Serial sections of 5 μm were obtained. The tissues were pre-treated with 10 μg/ml proteinase K (Sigma-Aldrich), and pre-hybridization was performed in the hybridization buffer (50% formamide, 5x SSC pH 4.5, 1% SDS, 500 μg/ml tRNA, 50 μg/ml heparin) in a water bath at 55 °C. Incubation with the antisense RNA probe was performed at 55 °C overnight, followed by incubation with 2% Boehringer blocking reagent (BBR)/MABT (0.2 M maleic acid, 0.3 M NaCl, 0.4 M NaOH, 0,1% Tween 20, DEPC-treated water), followed by another incubation with an anti-DIG-AP (Roche) antibody at 1:2000 in 2% BBR/MABT. Probe detection was performed after washing the samples in MABT, NTMT (5 M NaCl, Tris pH 9.5, 2 M MgCl_2_, 0.1% Tween 20, 1 M levamisole, DEPC-treated water), and NTMT with 20 μl/ml NBT/Bcip (nitro-blue tetrazolium/5-bromo-4-chloro-3-indolyl phosphate; Roche). The slides were mounted with 80% glycerol in PBS. The hybridized sections were photographed under a BX-41 Olympus microscope using a Q-Color 3/Olympus capture system.

### Total RNA extraction and RNA-seq sequencing

After euthanisation, the animal samples were macerated and homogenized in *TRIzol Reagent®* (Invitrogen) using Dispersing Tools S10 N-10G (IKA WERKE). *TRIzol Reagent®* (Invitrogen) was used at 1 ml of *TRIzol* per 100 mg of tissue. The quality of the isolated total RNA was evaluated in a Bioanalyzer (Agilent). RNA integrity numbers (RINs) ranging from 8 to 10 were considered suitable for RNA-seq.

For cDNA library construction, 2 μg of total RNA was treated with 1 U of *DNase I, Amplification Grade* (Invitrogen) and purified according to the Illumina protocol using magnetic microspheres for messenger RNA separation. The purified mRNA was fragmented in Illumina buffer. *SuperScript II* (Invitrogen) and oligo(dT) were used for reverse transcription of the first cDNA strand. The second cDNA strand was synthesized using *RNase H* and *DNA Polymerase I* (Illumina). The ends of the molecules were treated with *T4 DNA Polymerase* and *Klenow DNA Polymerase* (Illumina). The 3′ end of the synthesized cDNA were phosphorylated with *T4 PNK* (Illumina) and adenylated with *Klenow exo* (Illumina). Adaptors were added to the cDNA ends, and the samples were purified and selected by size (200 bp ±25 bp) through agarose gel electrophoresis (*QIAquick Gel Extraction Kit*, QIAGEN). The purified cDNA was quantified by RT-qPCR using adaptor-specific oligonucleotides (Illumina).

RNA-seq sequencing was performed using the *HiSeq 2500* platform (Illumina) according to the recommendations of the manufacturer and the paired-end reads protocol. Each sample was sequenced until reaching approximately 5 million reads/library.

### Transcriptome assembly and annotation

The *D. albiventris* transcriptome was assembled using a de novo approach. The raw data were trimmed to remove adaptors, sequencing artefacts, and low-quality fragments using the *NGS QC Toolkit* [126] and a Phred quality score of 15. High-quality reads were subjected to in silico normalization prior to de novo assembly. For normalization, decreasing sequencing coverage of highly represented regions with a fragment density greater than 30x sequencing coverage was considered. The normalized data were subjected to de novo transcriptome assembly using the *Trinity* tool [127]. In the assembly process, a minimum fragment overlap of 35 bp was considered. Only contigs longer than 300 bp were included within the assembled transcriptome for further analysis, which involved the following approaches: (i) differential gene expression analysis; (ii) gene ontology (GO) sequence annotation using *Blast2GO* [128]; and *KAAS* [129, 130].

### Differential gene expression analysis

For gene expression analysis, the assembled transcriptome was used as a gene model reference. All the raw RNA-seq data from the libraries were used in this step following data quality filtering and read alignment.

The libraries for each postnatal stage were separately aligned to the assembled reference transcriptome using the default parameters of *Bowtie* software [131] and then labelled together as biological replicates for statistical analysis.

A count matrix was constructed to represent the absolute number of aligned reads for each assembled transcript, with rows representing transcripts and columns representing fragment counts for a specific sample. The data distribution was fit according to a negative binomial distribution method, and a false discovery rate (FDR) correction [132] of 5% was then applied to control false-positive significance of transcript expression variation.

Statistical analysis was performed using the *DESeq2* package of *R/Bioconductor* [133]. Differential expression between developmental stages was detected for those transcripts with statistical significance (*p* < 0.05). Differentially expressed (DE) genes were analysed with *Blast2GO* for GO annotation [128]. Quantitative differences in differentially expressed transcripts between the developmental stages were represented in a Venn diagram to identify common or exclusive transcripts among stages. A transcript was considered “exclusive” if its expression was detected in only one stage of development among those analysed in this work. The upregulated [log_2_(fold change) > 1] and downregulated [log_2_(fold change) < − 1] genes between each pair of stages were also represented in Venn diagrams. The Venn diagram images were generated using Venny 2.1 [133].

The count matrix was used to calculate and generate a Euclidean distance matrix for hierarchical sample clustering, grouping samples according to the most similar transcriptome profiles. This method was used to generate a colourmap matrix representing how the sequenced samples (biological replicates) were correlated. The normalized count matrix was also employed to perform PCA (principal component analysis) using the *R* library *ggplots* from *DEseq/Bioconductor*. Additionally, a single linkage method was used to generate a dendrogram and a colourmap matrix to correlate all sample expression profiles according to colour, ranging from green (most different) to red (identical profiles).

A table of transcripts that were simultaneously expressed at the three stages was used to calculate and generate a matrix containing the expression levels of each transcript in each developmental stage. Additionally, a single linkage method was used to generate a dendrogram and a colourmap matrix to correlate the expression levels according to colour, ranging from green (lower expression values) to red (higher expression values). The colourmap matrix was obtained using the *d3heatmap* package of RStudio v3.4.1.

### Coverage between marsupial amino acid sequences

The Basic Local Alignment Search Tool (BLAST) X (NCBI BLAST+, available at ftp://ftp.ncbi.nlm.nih.gov/blast/executables/blast+/LATEST/) with default parameters was used to compare the nucleotide sequences of the *D. albiventris* transcripts to the amino acid sequences of *M. domestica* (available at ftp://ftp.ncbi.nlm.nih.gov/genomes/all/GCF/000/002/295/GCF_000002295.2_MonDom5/GCF_000002295.2_MonDom5_protein.faa.gz). The *D. albiventris* nucleotide sequences were translated into amino acid sequences to verify how many and which proteins of *M. domestica* correspond to those of *D. albiventris*. Sequences of *D. albiventris* showing 20 to 50% coverage identity to the *M. domestica* sequences were evaluated. The probability density graphics of the coverage identity were generated in the *gplots* and *ggplot2* packages of RStudio software.

### Coverage between marsupial nucleotide sequences

The coverage between the nucleotide sequences of *D. albiventris* and other marsupial species (*M. domestica*, *S. harrisii*, *P. cinereus*, and *M. eugenii*) was evaluated with the Basic Local Alignment Search Tool (BLAST) N (NCBI BLAST+, available at ftp://ftp.ncbi.nlm.nih.gov/blast/executables/blast+/LATEST/). This analysis was performed between the assembled skunk transcriptome and the assembled transcriptome available in the *Monodelphis domestica* NCBI database (ftp://ftp.ncbi.nlm.nih.gov/genomes/all/GCF/000/002/295/GCF_000002295.2_MonDom5/GCF_000002295.2_MonDom5_rna.fna.gz) and those of *Sarcophilus harrisii* (ftp://ftp.ncbi.nlm.nih.gov/genomes/all/GCF/000/189/315/GCF_000189315.1_Devil_ref_v7.0/GCF_000189315.1_Devil_ref_v7.0_rna.fna.gz) and *Phascolarctos cinereus* (ftp://ftp.ncbi.nlm.nih.gov/genomes/all/GCF/002/099/425/GCF_002099425.1_phaCin_unsw_v4.1/GCF_002099425.1_phaCin_unsw_v4.1_rna.fna.gz). For *Macropus eugenii,* we used the genome sequence (ftp://ftp.ncbi.nlm.nih.gov/genomes/all/GCA/000/004/035/GCA_000004035.1_Meug_1.1/GCA_000004035.1_Meug_1.1_genomic.fna.gz), as its transcriptome is not available. The probability density graphics of the coverage identity were generated in the *gplots* and *ggplot2* packages of RStudio software.

### RT-qPCR

cDNA was synthesized using 1 μg of total RNA following the recommendations of the *RevertAid™ H Minus First Strand cDNA Synthesis* kit (Fermentas). Diluted cDNAs (1:10 v/v) and *SYBR® Green Master Mix* (Bio-Rad) were used for the quantification of target genes via the quantitative reverse transcription polymerase chain reaction (RT-qPCR) approach in a *Rotor-Gene 3000* system (Corbett Research). Fluorescence was measured at the end of extension, providing the cycle threshold (C_T_) values. All reactions, including those with a template and omitting the template (negative controls), were run in biological triplicates (three biological samples for each developmental stage – P0, P5 and P10). The four evaluated genes of interest (GOI) followed by their forward (F) and reverse (R) primers in the 5′ to 3′ direction on the sense strand are as follows: HMGB3 (TCCATTATTACACAAACCAAGCA; GGAGGAAGAGGAGGAAGAGG), CRYGB (CATGGGCCATCAGTATTACC; GGAGCGTATTTCATTCATGTG), APOH (ATGAGCCAGGGGAACAAA; TTCACAGTATTAGGGTAGTCAAAGG) and HBZ (GCCCTCGTCCTCACCATCT; TGACAGCATCTCCAATAGCAC). The UBC gene was used as a reference gene (CACTTGGTGCTGCGTCTTC; TGCCTCTTTATTTGACCTTCTTC). The oligonucleotides were designed to amplify an ~ 200 bp amplicon from within different exons following the Minimum Information for Quantitative RT-PCR Experiments (MIQE) guidelines [134]. The applied cycling parameters were as follows: 50 °C for 2 min, 95 °C for 2 min, followed by 40 cycles of 95 °C for 15 s, 60 °C for 30 s and 72 °C for 20 s.

The analysis of differential gene expression was performed using REST 2009 (*Relative Expression Software Tool*, V.2.0.13) software via randomization tests *(Pair Wise Fixed Reallocation Randomization Test©*) [135] with 95% significance.

### DIAMOND and BLASTP analyses

DIAMOND v0.9.19 (double index alignment of next-generation sequencing data) [136] was used to align the assembled transcripts against the NCBI-nr database. The following parameter settings were used for the program: -e 0.00001 -k 1 --masking 0 --matrix blosum62 --gapopen 11 --gapextend 1 --more-sensitive --salltitles –sallseqid. The results returned only the hits with the best score for each opossum transcript.

All DIAMOND subject IDs were listed without redundancies and used as the protein database input in the NCBI tool Batch Entrez (https://www.ncbi.nlm.nih.gov/sites/batchentrez). The output was used as the input for BLASTP (Basic Local Alignment Search Tool) analysis, using the NCBI BLAST+ tool (ftp://ftp.ncbi.nlm.nih.gov/blast/executables/blast+/LATEST/) together with the *M. domestica* amino acid sequences (ftp://ftp.ncbi.nlm.nih.gov/genomes/all/GCF/000/002/295/GCF_000002295.2_MonDom5/GCF_000002295.2_MonDom5_protein.faa.gz). The following parameter settings were used for the program: -evalue 1e-5 -max_target_seqs 1. Thus, we obtained UniProt Entry IDs (using Retrieve ID tool; http://www.UniProt.org/uploadlists/) for further ClueGO analysis (Additional file 3). The grey short-tailed opossum was selected for further analysis using GO enrichment networks, as data for this species are available in ClueGO, and the DIAMOND results showed the greatest number of hits for *M. domestica*.

### GO pathway enrichment analysis

The plugin ClueGO (v.2.3.5) [137] for Cytoscape (v3.4.0) [138] was used for GO enrichment analysis. The node colours represent the functional groups. The node size represents the term enrichment significance. The edges connecting the nodes were based on the Kappa statistic (Kappa score threshold of 0.4). Only the most significant term in the group was labelled. The right-sided hypergeometric test was used to identify overrepresented GO terms, and the Benjamini-Hochberg method was used for the correction of *p*-values (*p* < 0.05).

## Supplementary information

**Additional file 1: **Probability density values for the coverage identity of the *D. albiventris* vs *M. domestica* sequence alignment. A coverage of 30% is more likely to be associated with higher identity values and a less likely to be associated with lower identity values. Min: Minimum. 1st Qu: first quantile. 3rd Qu: third quantile. Max: maximum.

**Additional file 2: **Probability density values for BLASTN analysis between *D. albiventris* and other marsupial species. Probability density values between *D. albiventris* and other marsupial species (*M. domestica*, *S. harrisii*, *P. cinereus* and *M. eugenii*) determined with BLASTN. According to the analysis of the median and mean values, the coverage percentages for which there is a greater probability of presenting higher identity values and a lower tendency to present lower identity values (bold text) are as follows: 90% for *M. domestica* and *S. harrisii*; 20% for *P. cinereus* and *M. eugenii*. Min: Minimum. 1st Qu: first quantile. 3rd Qu: third quantile. Max: maximum.

**Additional file 3:** DIAMOND results for the assembled opossum transcripts. The data are presented in seven sheets: all results for the three stages (“total”), all results for each pair of stages (“P0xP5”, “P0xP10” and “P5xP10”), differentially expressed results for each pair of stages (“P0xP5_DE”, “P0xP10_DE” and “P5xP10_DE”), and differentially expressed genes exclusively expressed in each stage (“P0_DE_exclusive”; “P5_DE_exclusive”; “P10_DE_exclusive”).

**Additional file 4: **RT-qPCR validation of expression profiles for selected genes. The RNA-seq expression results for four selected genes (HMGB3, CRYGB, APOH and HBZ) were confirmed by RT-qPCR. Relative expression was evaluated at the later stage in relation to the early stage (red line): expression at P5 in relation to P0 (**A**), expression at P10 in relation to P0 (**B**), and expression at P10 in relation to P5 (**C**). The expression values (C_T_) were normalized against the reference gene UBC. The boxed area in a whisker-box plot encompasses 50% of all observations, the dotted line represents the sample median, and the whiskers represent the outer 50% of observations. *p < 0.05. The results were analysed using REST 2009 software.

**Additional file 5: **P0, P5 and P10 hybridized tissues for titin. Sagittal view of *D. albiventris* hybridized tissues for titin mRNA at P0 (A to D), P5 (E and F), and P10 (G to J). Diaphragm (A, E and H). Tongue (B and C). Ventral skeletal muscle (D). Longitudinal and transverse views of limb skeletal muscle (F). Sagittal view of the heart (G). Frontal view of the tongue and mandible skeletal muscle (I). Frontal view of the eye (J). ClueGO networks for overrepresented GO functional groups related to muscle based on the DE transcripts between P0 and P5 (K). *Asterisk,* Meckel’s cartilage; *at*, atrium; *ca*, cartilage; *d*, diaphragm; *em*, extrinsic muscle of eye; *it*, small intestine; *li*, liver; *ln*, eye lens; *lsm*, longitudinal view of skeletal muscle; *lu*, lungs; *mm*, mandible skeletal muscle; *pd*, periderm; *se*, heart septum; *sm*, skeletal muscle; *st*, stomach; *to*, tongue; *tsm*, transversal view of skeletal muscle; *ve*, ventricle. *Bar scale*, 50 μm (C, D); 150 μm (A, F, I); 300 μm (B, E, G, J); 650 μm (H).

**Additional file 6:** Enriched GO terms obtained through ClueGO analysis. Gene names and GO terms of biological processes for enriched transcripts analysed with ClueGO. The data are presented in eight sheets: all results for the three stages (“total”), differentially expressed results for the three stages (“total DE”), differentially expressed results for P0xP5 (“P0xP5 DE”), differentially expressed results for downregulated transcripts at P5 in relation to P0 [“P5(P0) DE down”], differentially expressed results for upregulated transcripts at P5 in relation to P0 [“P5(P0) DE up”], differentially expressed results for P5xP10 (“P5xP10 DE”), differentially expressed results for P5xP10 (“P5xP10 DE”), differentially expressed results for downregulated transcripts at P10 in relation to P5 [“P5xP10 DE down”], differentially expressed results for upregulated transcripts at P10 in relation to P5 [“P5xP10 DE up”].

## Data Availability

The raw transcriptome sequencing data (RNA-seq) from the biological replicates and developmental stages of *Didelphis albiventris* are available under the NCBI-BioProject submission code PRJNA544055. The datasets supporting the conclusions of this article are included within the article and its additional files.

## Abbreviations

AIPL1: Aryl Hydrocarbon Receptor Interacting Protein Like 1
Anti-DIG-AP: anti-digoxigenin antibody, conjugated with alkaline phosphatase
APOH: Apolipoprotein H
BBR: Boehringer Blocking Reagent
Bcip: 5-bromo-4-chloro-3′-indolyphosphate
BLAST: Basic Local Alignment Search Tool
BLASTN: Search nucleotide databases using a nucleotide query
BLASTP: Search protein databases using a protein query
BLASTX: Search protein databases using a translated nucleotide query
bp: Base pair
cDNA: Complementary DNA
CNS: Central nervous system
CRYBA1: Crystallin Beta A1
CRYBA4: Crystallin Beta A4
CRYBB1: Crystallin Beta B
CRYBB2: Crystallin Beta B2
CRYGB: Crystallin Gamma B
C_T_: Cycle threshold
DE: Differentially expressed
DEPC: Diethyl pyrocarbonate
DIAMOND: Double index alignment of next-generation sequencing data
EKLF: Erythroid Kruppel-Like Factor
EMP: Erythro-myeloid progenitor
F: Forward primer
FDR: False Discovery Rate
FGF4: Fibroblast Growth Factor 4
GJC1: Gap Junction Protein Gamma 1
GO: Gene ontology
GOI: Genes of interest
GPR37L1: G Protein-Coupled Receptor 37 Like 1
HBZ: Hemoglobin subunit Zeta
HES1: Hairy and Enhancer of Split 1
HMGB3: High Mobility Group Box 3
ID: Identifier
IgA: Immunoglobulin A
IRX1: Iroquois transcription factor
ISH: In situ hybridization
KLF1: Kruppel Like Factor 1
MABT: Maleic acid buffer containing Tween 20
MEP: Megakaryocyte-erythrocyte progenitor
MGAT3: Mannosyl (Beta-1,4-)-Glycoprotein Beta-1,4-N-Acetylglucosaminyltransferase
MIQE: Minimum Information for Quantitative RT-PCR Experiments
MOGAT3: Monoacylglycerol O-Acyltransferase 3
mRNA: Messenger RNA
MSX1: Msh Homeobox 1
NBT: Nitro-blue tetrazolium
NCBI: National Center for Biotechnology Information
NGS: Next Generation Sequencing
NHE4: Na^+^/H^+^ Exchanger 4
nr: Non-redundant
NST: Non-shivering thermogenesis
NTMT: NaCl, Tris-HCl, MgCl2, Tween 20
oligo(dT): Deoxythymine nucleotides
P: Postnatal stage
p, or *p*-value: Probability value, or significance value
PBS: Phosphate-buffered saline
PCA: Principal Component Analysis
PFA: Paraformaldehyde
PTK7: Tyrosine-Protein Kinase-Like 7
R: Reverse primer
REST: Relative Expression Software Tool
RIN: RNA integrity number
RNA: Ribonucleic acid
RNA-seq: RNA sequencing
RT: Room temperature
RT-qPCR: Quantitative reverse transcription polymerase chain reaction
SD: Standard deviation
SDS: Sodium dodecyl sulfate
SLC9A4: Solute Carrier Family 9 Member A4
SOBP: Sine Oculis Binding Protein Homolog
SSB: S-shaped body
SSC: Saline-sodium citrate
tRNA: Transfer RNA
UBC: Ubiquitin C
UTP: Uridine-5′-triphosphate
v/v: Volume/volume
