# Supplementary material for: Didelphis albiventris: an overview of unprecedented transcriptome sequencing of the white-eared opossum
Source: BMC Genomics. 2019 Nov 15;20:866. doi: 10.1186/s12864-019-6240-x (PMC6858782; doi:10.1186/s12864-019-6240-x)
Supplement: Supplementary file 1 — Additional file 1: Probability density values for the coverage identity of the D. albiventris vs M. domestica sequence alignment. A coverage of 30% is more likely to be associated with higher identity values and a less likely to be associated with lower identity values. Min: Minimum. 1st Qu: first quantile. 3rd Qu: third quantile. Max: maximum. [file 12864_2019_6240_MOESM1_ESM.docx]

| **Coverage** | **Min** | **1st Qu** | **Median** | **Mean** | **3rd Qu** | **Max** |
| --- | --- | --- | --- | --- | --- | --- |
| 20% | 16,41 | 33,06 | 44,23 | 45,87 | 56,40 | 100,00 |
| 30% | 16,59 | 38,32 | 53,55 | 51,19 | 61,11 | 100,00 |
| 40% | 17,69 | 29,73 | 36,02 | 38,96 | 47,35 | 81,88 |
| 50% | 24,33 | 30,10 | 36,02 | 36,03 | 42,86 | 63,45 |
