# Supplementary material for: Didelphis albiventris: an overview of unprecedented transcriptome sequencing of the white-eared opossum
Source: BMC Genomics. 2019 Nov 15;20:866. doi: 10.1186/s12864-019-6240-x (PMC6858782; doi:10.1186/s12864-019-6240-x)
Supplement: Supplementary file 2 — Additional file 2: Probability density values for BLASTN analysis between D. albiventris and other marsupial species. Probability density values between D. albiventris and other marsupial species (M. domestica, S. harrisii, P. cinereus and M. eugenii) determined with BLASTN. According to the analysis of the median and mean values, the coverage percentages for which there is a greater probability of presenting higher identity values and a lower tendency to present lower identity values (bold text) are as follows: 90% for M. domestica and S. harrisii; 20% for P. cinereus and M. eugenii. Min: Minimum. 1st Qu: first quantile. 3rd Qu: third quantile. Max: maximum. [file 12864_2019_6240_MOESM2_ESM.docx]

| ***M. domestica*** | | | | | | | | | | | | |  | ***S. harrisii*** | | | | | | |
| --- | --- | --- | --- | --- | --- | --- | --- | --- | --- | --- | --- | --- | --- | --- | --- | --- | --- | --- | --- | --- |
| **Coverage** | **Min.** | **1st Qu.** | | **Median** | | **Mean** | | | **3rd Qu.** | | **Max.** | |  | **Coverage** | **Min.** | **1st Qu.** | **Median** | **Mean** | **3rd Qu.** | **Max.** |
| 20% | 68.92 | 81.76 | | 89.19 | | 88.16 | | | 95.02 | | 100.00 | |  | 20% | 69.54 | 81.61 | 88.16 | 86.98 | 92.47 | 100.00 |
| 30% | 68.92 | 82.02 | | 90.27 | | 88.61 | | | 95.40 | | 100.00 | |  | 30% | 69.54 | 82.26 | 88.55 | 87.24 | 92.63 | 100.00 |
| 40% | 68.92 | 83.33 | | 91.47 | | 89.36 | | | 95.76 | | 100.00 | |  | 40% | 69.54 | 83.11 | 88.88 | 87.50 | 92.78 | 100.00 |
| 50% | 68.92 | 84.01 | | 91.83 | | 89.70 | | | 95.84 | | 100.00 | |  | 50% | 69.54 | 83.44 | 89.00 | 87.59 | 92.81 | 100.00 |
| 60% | 69.77 | 84.31 | | 92.02 | | 89.88 | | | 95.88 | | 100.00 | |  | 60% | 69.54 | 83.73 | 89.15 | 87.74 | 92.88 | 100.00 |
| 70% | 69.97 | 84.82 | | 92.31 | | 90.15 | | | 95.94 | | 100.00 | |  | 70% | 69.60 | 84.10 | 89.33 | 87.96 | 93.03 | 100.00 |
| 80% | 69.97 | 85.76 | | 92.62 | | 90.54 | | | 96.04 | | 100.00 | |  | 80% | 69.89 | 84.41 | 89.56 | 88.14 | 93.20 | 100.00 |
| **90%** | **69.97** | **87.00** | | **92.99** | | **91.04** | | | **96.17** | | **100.00** | |  | **90%** | **70.18** | **84.98** | **89.93** | **88.51** | **93.43** | **100.00** |
| 100% | 70.02 | 87.42 | | 92.90 | | 91.33 | | | 96.11 | | 100.00 | |  | 100% | 69.54 | 81.82 | 88.17 | 87.13 | 92.54 | 100.00 |
|  | | |  | |  | |  |  | |  | |  |  |  |  |  |  |  |  |  |
| ***P. cinereus*** | | | | | | | | | | | | |  | ***M. eugenii*** | | | | | | |
| **Coverage** | **Min.** | **1st Qu.** | | **Median** | | **Mean** | | | **3rd Qu.** | | **Max.** | |  | **Coverage** | **Min.** | **1st Qu.** | **Median** | **Mean** | **3rd Qu.** | **Max.** |
| **20%** | **69.19** | **78.86** | | **81.82** | | **82.14** | | | **85.19** | | **100.00** | |  | **20%** | **69.73** | **79.33** | **82.76** | **82.91** | **86.32** | **100.00** |
| 30% | 69.19 | 77.33 | | 79.05 | | 79.58 | | | 81.43 | | 100.00 | |  | 30% | 69.73 | 77.54 | 80.54 | 81.16 | 84.04 | 100.00 |
| 40% | 69.19 | 76.17 | | 78.01 | | 78.83 | | | 80.86 | | 100.00 | |  | 40% | 69.73 | 76.53 | 79.20 | 80.31 | 82.98 | 100.00 |
| 50% | 69.19 | 75.91 | | 77.59 | | 78.47 | | | 80.22 | | 100.00 | |  | 50% | 69.73 | 76.10 | 78.68 | 80.04 | 82.68 | 100.00 |
| 60% | 69.19 | 75.76 | | 77.32 | | 78.27 | | | 79.84 | | 100.00 | |  | 60% | 69.73 | 75.85 | 78.49 | 79.98 | 82.76 | 100.00 |
| 70% | 69.19 | 75.51 | | 76.92 | | 78.03 | | | 79.02 | | 100.00 | |  | 70% | 69.73 | 75.84 | 78.59 | 80.12 | 83.18 | 100.00 |
