# Supplementary figures and images for: Didelphis albiventris: an overview of unprecedented transcriptome sequencing of the white-eared opossum
Source: BMC Genomics. 2019 Nov 15;20:866. doi: 10.1186/s12864-019-6240-x (PMC6858782; doi:10.1186/s12864-019-6240-x)

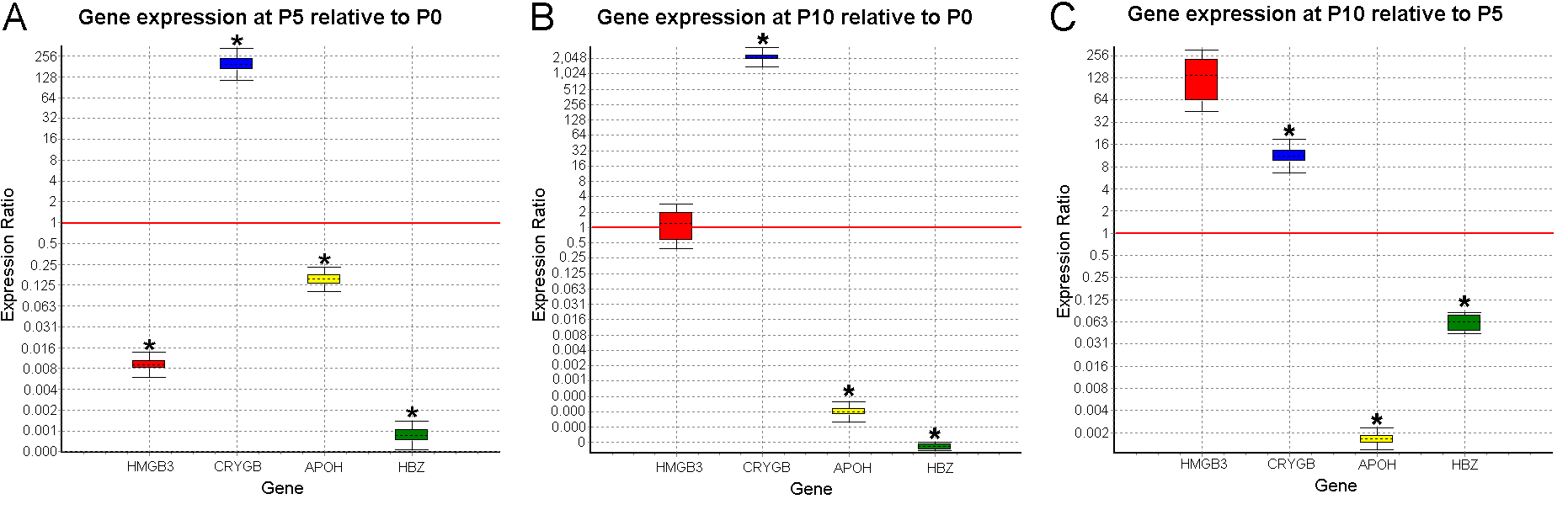

Supplement: Supplementary file 4 — Additional file 4: RT-qPCR validation of expression profiles for selected genes. The RNA-seq expression results for four selected genes (HMGB3, CRYGB, APOH and HBZ) were confirmed by RT-qPCR. Relative expression was evaluated at the later stage in relation to the early stage (red line): expression at P5 in relation to P0 (A), expression at P10 in relation to P0 (B), and expression at P10 in relation to P5 (C). The expression values (CT) were normalized against the reference gene UBC. The boxed area in a whisker-box plot encompasses 50% of all observations, the dotted line represents the sample median, and the whiskers represent the outer 50% of observations. *p < 0.05. The results were analysed using REST 2009 software. [file 12864_2019_6240_MOESM4_ESM.tif]

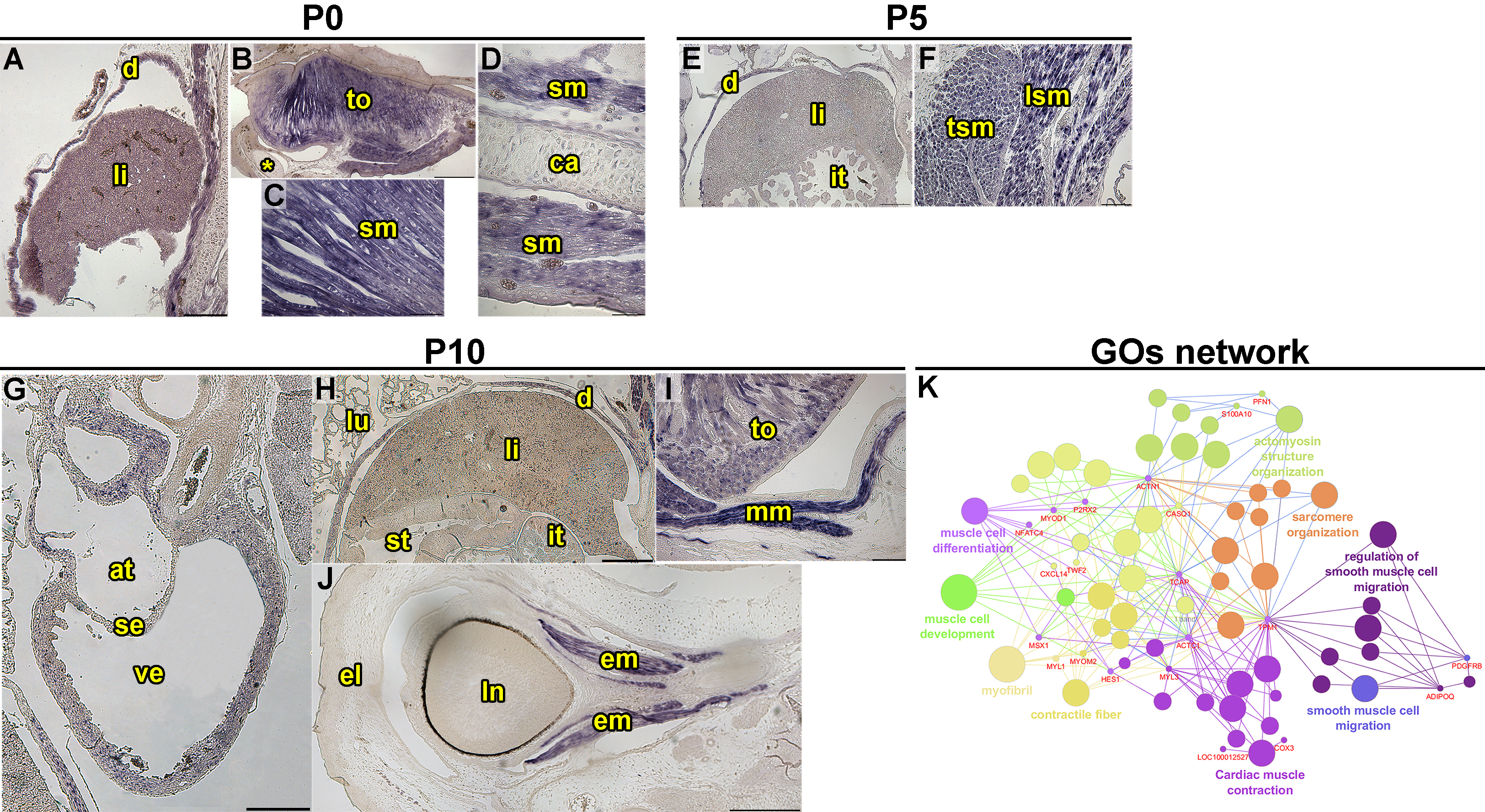

Supplement: Supplementary file 5 — Additional file 5: P0, P5 and P10 hybridized tissues for titin. Sagittal view of D. albiventris hybridized tissues for titin mRNA at P0 (A to D), P5 (E and F), and P10 (G to J). Diaphragm (A, E and H). Tongue (B and C). Ventral skeletal muscle (D). Longitudinal and transverse views of limb skeletal muscle (F). Sagittal view of the heart (G). Frontal view of the tongue and mandible skeletal muscle (I). Frontal view of the eye (J). ClueGO networks for overrepresented GO functional groups related to muscle based on the DE transcripts between P0 and P5 (K). Asterisk, Meckel’s cartilage; at, atrium; ca, cartilage; d, diaphragm; em, extrinsic muscle of eye; it, small intestine; li, liver; ln, eye lens; lsm, longitudinal view of skeletal muscle; lu, lungs; mm, mandible skeletal muscle; pd, periderm; se, heart septum; sm, skeletal muscle; st, stomach; to, tongue; tsm, transversal view of skeletal muscle; ve, ventricle. Bar scale, 50 μm (C, D); 150 μm (A, F, I); 300 μm (B, E, G, J); 650 μm (H). [file 12864_2019_6240_MOESM5_ESM.tif]
